# Supplementary material for: How migrants’ transcultural perceptions shape their children’s bilingual language development: Insights from a cross-sectional multicultural study
Source: PLoS One. 2025 Oct 17;20(10):e0317645. doi: 10.1371/journal.pone.0317645 (PMC12533872; doi:10.1371/journal.pone.0317645)
Supplement: S1 File — (DOCX) [file pone.0317645.s001.docx]

**S1 File: Rating grid development, testing, and application processes**

Diagram inspired by [50]. We developed a transcultural perception measurement tool in close consultation with experts in transcultural clinics who have been working in this field for over 10 years and who have experience with first- and second-generation migrants and knowledge of the parents' regions of origin. This tool includes: i) a semistructured interview guide for the investigators who interviewed parents about transcultural/familial factors; ii) for each factor, a Likert-type numerical value scoring grid for the raters who quantitized the qualitative corpus made up of parent’ narrative answers. Value range scores obtained by quantitization had direction and magnitude representing the intensity of the factor (ranging from -5 to 5): the more positively the parent perceived a factor, the higher the score. For each variable, anchor points were operationalized by a specific statement that best exemplified that specific level of intensity. For example, concerning the factor transgenerational transmissions, the statement descriptive of a rating -5 is: “*the subject is at odds with transmission, which may be rejected or represent an obstacle for the subject. The question of transmission can be associated with insecurity and abandonment. Transgenerational transmission is not encouraged and is blocked*.” The statement descriptive of a rating ‘5’ was “*the participant values this transmission, writing himself and his child in a filiation; cultural transmission appears direct and intense, with strong intention*”. The full scoring-grid is included in appendix S2.
