# Supplementary material for: How migrants’ transcultural perceptions shape their children’s bilingual language development: Insights from a cross-sectional multicultural study
Source: PLoS One. 2025 Oct 17;20(10):e0317645. doi: 10.1371/journal.pone.0317645 (PMC12533872; doi:10.1371/journal.pone.0317645)
Supplement: S2 File — (DOCX) [file pone.0317645.s002.docx]

MRC: Migration-related change

EFR: Extended-family relationships

TGT: Transgenerational transmission:
 insertion in the family line

The scores must be based on the parents' explicit statements during the interview. It is important to focus on the particular question asked. To avoid being influenced by other factors for the score of a specific variable, do not read the whole interview before starting to score it.

# Perception of migration-related change

| **Interview guide:**  *What do you think of the changes in your life since you arrived in France?*  Let the parent talk and then have him/her specify the items considered important  *You said: " _ _ _ _ _ ." Can you be more specific? Can you give me an example that illustrates what you just said?* |
| --- |

**Instructions to scorers:**

This involves the perception of a transformation related to the migration situation between their arrival in the host country and today. **The valence of the perception of change is what is to be measured**, regardless of the aspect mentioned.

The perception is independent of the intensity of the change. The change may be modest but psychically invested.

Negative perception: During the interview, the subject experienced the change as undesirable and rejects it. The subject withdrew into premigration experiences, with a single reference exclusively to what he or she has known for a long time. The subject was closed off to what might change or experienced the change as something negative.

**Neutral** perception: At the moment of the interview, the subject was indifferent to the change. Indifference is coded 0.

**Positive** perception: During the interview, the subject perceived the change as an opening, a discovery that is valued. The subject has a taste for diversity and made several references to it.

-5 -4 -3 -2 -1 0 1 2 3 4 5

🡸 ----------------------------------------------------------------------- 🡺

Negative perception Positive perception

|  |  |  |  |  |  |
| --- | --- | --- | --- | --- | --- |
|  |  |  | Score: |  |  |
|  | Scorable^1^: |  |  |  |  |
|  |  |  |  |  |  |

# ^1^ The subject's responses could be scored: yes/no

# Perception of extended-family relationships

| **Interview guide:**  *What members of your family do you see? Parents, grandparents, brothers and sisters, aunts and uncles, cousins etc.)?* *You may take your time to answer to answer.*  Reminders:  *• Do you see your family here?*  *• Do you see your family in your native country?*  *•* If the person answered that she sees no one: *Do you get any news from or about your family?*  *How are things with your family: parents, grandparents, brothers and sisters, aunts and uncles, cousins, etc.)? Can you tell me about your relationships with your family?*  Let them talk and then:  *Are things as you would like them to be?*  Reminder: *You said: " _ _ _ _ _ ." Can you be more specific? Can you give me an example that illustrates what you just said?* |
| --- |

**Instructions to scorers:**

Here we are considering not the subject's nuclear family — their spouse and children — but their extended family. This means the relationships of each parent, as described by the interviewee, with their own parents, brothers and sisters, uncles, aunts and cousins, whether or not they live together and whether or not the latter live in France.

The score should not differentiate between the family in France and in the country they migrated from.

The valence of the perception of change is what is to be measured. The perception is independent of the frequency. The relationships may be almost absent but still psychically invested.

**Negative** perception**:** During the interview, the subject mentioned conflictual relationships with extended family members, whether or not they are in contact. The subject associated these relationships with experiences of disapproval or unpleasant feelings.

**Neutral** perception: During the interview, the subject did not appear invested in these family relationships and suggested some indifference to or distancing or separation from the family.

**Positive** perception: During the interview, the parent mentioned these harmonious relationships, experienced as support. The parent sought contacts with them, that is, contacts associated with the experience of pleasure. The subject expressed feelings of missing these relatives when they were not present or due to how far away they live.

-5 -4 -3 -2 -1 0 1 2 3 4 5

🡸 ----------------------------------------------------------------------- 🡺

Negative perception Positive perception

|  |  |  |  |  |  |
| --- | --- | --- | --- | --- | --- |
|  |  |  | Score: |  |  |
|  | Scorable^1^: |  |  |  |  |
|  |  |  |  |  |  |

# Perception of transgenerational transmission: insertion in the family line

| **Interview guide:**  *How is your relationship with your child? You can take your time to answer*  Reminder: *You said: " _ _ _ _ _ ." Can you tell us about this relationship? Can you give me an example that illustrates what you just said?*  *What has your child taken from his maternal or paternal grandparents? (Physical resemblance, or similar character or values…)*  *What have you taken from your parents that you have taught your child?*  *How did you choose his or her first name?*  *Does the relationship you have with your child resemble the one you had with your parents?* |
| --- |

**Instructions to scorers:**

By transgenerational transmission, we mean inscription/insertion in the family line, that is the transmission of family's culture and heritage between grandparents, parents, and children, as described by the parent.

This perception score must assess the allegiance in the transmission, that is, how much the parent wish to place their child into their family line.

Negative perception During the interview, the subject was opposed to transmission, which he or she may reject or consider an obstacle. The issue of transmission can be related to an experience of insecurity or even abandonment. Transgenerational transmission is not encouraged, is even blocked.

Neutral perception: During the interview, the subject expressed indifference about this question. It should be scored as 0.

Positive perception:

Moderate: During the interview, the subject valued transmission and placed himself and the child in the family line, moderately intentionally. It should be scored from 1 to 3.

Strong: The transmission is direct and intense, and strongly intentional. It should be scored from 4 to 5.

The perception can be positive even though what is transmitted to the child is not identical to the transmission received from the subject's parents. It does not matter if the transmission is not the same.

-5 -4 -3 -2 -1 0 1 2 3 4 5

🡸 ----------------------------------------------------------------------- 🡺

Negative perception Positive perception

|  |  |  |  |  |  |
| --- | --- | --- | --- | --- | --- |
|  | Scorable^1^: |  | Score: |  |  |
|  |  |  |  |  |  |
|  |  |  |  |  |  |
