# Supplementary material for: How migrants’ transcultural perceptions shape their children’s bilingual language development: Insights from a cross-sectional multicultural study
Source: PLoS One. 2025 Oct 17;20(10):e0317645. doi: 10.1371/journal.pone.0317645 (PMC12533872; doi:10.1371/journal.pone.0317645)
Supplement: S1 Table — Estimates are beta coefficients for linear regression (Models A, B, C, and D) and odds ratios (Model E) for logistic regressions with 95% confidence intervals. a Model Ma3 adjusted for ELAL Expression scale score, Mb3 adjusted for ELAL Comprehension scale score, Mc3 adjusted for N-EEL Expression scale score, and Md3 adjusted for N-EEL Comprehension scale score. No such adjustment for Me3 because ML storytelling skills were not scored with the N-EEL scale. (DOCX) [file pone.0317645.s003.docx]

**S1 Table. Full results of M3 multiple regression models**

|  | Model 3a | Model 3b | Model 3c | Model 3d | Model 3e |  |
| --- | --- | --- | --- | --- | --- | --- |
|  | **Outcome = N-EEL**  **Comprehension score** | **Outcome = N-EEL**  **Expression score** | **Outcome = ELAL**  **Comprehension score** | **Outcome = ELAL**  **Expression score** | **Outcome = ELAL**  **Storytelling (able to formulate a story yes/no)** |  |
| *Perception of MRC* |  |  |  |  |  |  |
| *Less favorable* | ref | ref | ref | ref | 1 |  |
| *Strongly positive* | -0.12 (-0.46, 0.22) | 0.01 (-0.36, 0.37) | -0.25 (-0.59, 0.10) | **-0.44 (-0.72, -0.15)** | **0.21 (0.04, 0.89)** |  |
| *No memory before migration* | 0.04 (-0.49, 0.57) | 0.17 (-0.39, 0.72) | 0.30 (-0.24, 0.85) | -0.01 (-0.47, 0.45) | 0.52 (0.04, 3.78) |  |
| *Perception of EFR* | | | | | | |
| *Less favorable* | ref | ref | ref | ref | 1 |  |
| *Strongly positive* | 0.01 (-0.30, 0.32) | 0.21 (-0.10, 0.52) | **-0.37 (-0.68, -0.07)** | -0.08 (-0.37, 0.18) | **0.25 (0.07, 0.82)** |  |
| *Perception of TGT* |  |  |  |  |  |  |
| *Less favorable* | ref | ref | ref | ref | 1 |  |
| *Strongly positive* | -0.10 (-0.41, 0.21) | -0.10 (-0.42, 0.23) | 0.20 (-0.11, 0.51) | **0.33 (0.08, 0.59)** | 2.64 (0,75, 10.57) |  |
| *Child's age* | **0.59 (0.36, 0.82)** | **0.66 (0.43, 0.90)** | 0.16 (-0.09, 0.41) | **0.32 (0.10, 0.53)** | **2.72 (1.10, 7.76)** |  |
| *Child's sex* |  |  |  |  |  |  |
| *Male* | ref | ref | ref | ref | 1 |  |
| *Female* | 0.22 (-0.08, 0.52) | 0.14 (-0.17, 0.45) | -0.20 (-0.51, 0.10) | 0.03 (-0.23, 0.28) | 2.48 (0.73, 9.44) |  |
| *Sibling rank* | | | | | | |
| *Rank in siblings >= 2* | ref | ref | ref | ref | 1 |  |
| *First child (Oldest)* | -0.16 (-0.49, 0.17) | -0.23 (-0.57, 0.12) | **0.37 (0.03, 0.71)** | 0.15 (-0.14, 0.44) | 4.16 (0.94, 22.55) |  |
| *Score measuring the equivalent skill in the other language ^a^* | **0.24 (0.05, 0.43)** | 0.00 (-0.24, 0.25) | **0.24 (0.04, 0.44)** | 0.00 (-0.17, 0.16) | nd |  |
| *Parents' highest occupational category* | | | | | | |
| *Other* | ref | ref | ref | ref | 1 |  |
| *Employee* | -0.18 (-0.54, 0.18) | **-0.45 (-0.83, -0.08)** | -0.14 (-0.51, 0.22) | -0.12 (-0.43, 0.20) | 2.55 (0.64, 11.18) |  |
| *Worker* | **-0.96 (-1.49, -0.43)** | **-0,99 (-1.54, -0.45)** | 0.15 (-0.43, 0.72) | 0.12 (-0.36, 0.60) | 2.61 (0,27, 36.87) |  |
| *Years lived in France* | 0.02 (0.00, 0.04) | 0.01 (-0.01, 0.03) | **-0.02 (-0.05, 0.00)** | **-0.02 (-0.04, 0.00)** | 0.98 (0.89, 1.07) |  |
| *Heritage language* | | | | | | |
| *Arabic* | ref | ref | ref | ref | 1 |  |
| *Soninke* | **-0.63 (-1.05, -0.22)** | -0,41 (-0.86, 0.05) | 0.02 (-0.41, 0.46) | **-0.61 (-0.97, -0.26)** | **0.14 (0.02, 0.69)** |  |
| *Tamil* | **-0.71 (-1.11, -0.32)** | -0.35 (-0.77, 0.07) | **0.68 (0.28, 1.09)** | **0.52 (0.19, 0.85)** | 3.16 (0.61, 19.27) |  |
| *Language used by the parent to the child* | | | | | | |
| *ML only* | ref | ref | ref | ref | 1 |  |
| *ML and HL* | -0.04 (-0.84, 0.76) | 0,09 (-0.74, 0.93) | -0.35 (-1.18, 0.47) | -0.31 (-1.00, 0.38) | 0,41 (0.01, 6.36) |  |
| *HL only* | -0,07 (-0.91, 0.78) | 0,13 (-0.75, 1.01) | -0.46 (-1.33, 0.41) | -0.25 (-0.97, 0.48) | 0,38 (0.01, 6.72) |  |
| *Language used by the child to the parent* | | | | | | |
| *ML only* | ref | ref | ref | ref | 1 |  |
| *ML and HL* | 0.15 (-0.22, 0.52) | -0,05 (-0.44, 0.34) | -0.09 (-0.47, 0.29) | -0.09 (-0.41, 0.22) | 1.05 (0.25, 4.05) |  |
| *HL only* | -0.48 (-1.06, 0.10) | **-0,81 (-1.41, -0.22)** | 0.08 (-0.51, 0.68) | -0.07 (-0.58, 0.44) | 1.05 (0.07, 18.50) |  |
| *Use of HL with siblings* | | | | | | |
| *No (No siblings or the siblings used only ML among themselves)* | ref | ref | ref | ref | 1 |  |
| *Use of ML and HL* | **-0.59 (-0.97, -0.20)** | -0,40 (-0.81, 0.01) | 0.33 (-0.07, 0.74) | **0.38 (0.05, 0.72)** | **HL: 6.25 (1.51, 31.24)** |  |
| *Use of HL only* | **-0.68 (-1.28, -0.08)** | -0,31 (-0.97, 0.34) | 0.60 (-0.02, 1.23) | **0.91 (0.39, 1.42)** | ML: 0.33 (0.02, 3.74) |  |
