# Supplementary material for: How migrants’ transcultural perceptions shape their children’s bilingual language development: Insights from a cross-sectional multicultural study
Source: PLoS One. 2025 Oct 17;20(10):e0317645. doi: 10.1371/journal.pone.0317645 (PMC12533872; doi:10.1371/journal.pone.0317645)
Supplement: Attachment — Submitted filename: REVIEWING PLOS ONE20250829_ Response.docx [file pone.0317645.s005.docx]

29/08/2025

Dear Dr. Ortega-Altamirano,

Thank you for your constructive comments on our manuscript and for the opportunity to submit a revision.

We attach the clean, revised manuscript and the version with the changes tracked. Our responses to the reviewers’ comments are provided below. We hope that the changes we have made address all your concerns and that manuscript describing innovative research in the field of children's language development in a multicultural society can now be published.

We also note that we have modified the title to make it easier and clearer for readers to understand. If you think it is too late to modify the title, we will understand.

Please do not hesitate to contact us if you have any further questions or suggestions.

We look forward to hearing from you at your earliest convenience.

Yours sincerely,

***Professor Marie Rose Moro***

- Professor at Paris Cité University and Research Director at the National Institute of Medical Research (CESP/INSERM).
- Head of Department at the Maison de Solenn (AP-HP, Hôpital Cochin, Paris, France) : www.maisondesolenn.fr
- Member of the French National Academy of Medicine.
- Member of the Institut Universitaire de France (IUF).
- Honorary doctorate from the University of Mons (Belgium).
- Scientific director of the transcultural journal *L’autre* (www.revuelautre.com)
- President of the International Association of Ethnopsychiatry (www.aiep-transculturel.com).

**Responses to the editor**

**I suggest to the authors to emphasize on the reasons for addressing the research question.**

**Response**:

Thank you for your question.

This study was initiated in response to clinical needs identified at our hospitals, located in Paris and its multicultural and multilingual suburbs. Multilingualism among children is an issue in many other cities in Europe and around the world. Existing language assessment tools are inappropriate for assessing the language abilities of bilingual migrant children as they often underestimate their skills due to the distributed and nonlinear nature of bilingual development.

To address this issue, our research team has previously developed several tools applying a transcultural methodology tailored to multicultural contexts. One is the Avicenne ELAL, a tool designed to evaluate heritage language skills acquired within the home. We also created a parent interview protocol to contextualize children’s language abilities by gathering data on family language practices and parental attitudes towards cultural and linguistic transmission.

The use of minority languages varies significantly according to family structure (nuclear versus extended), social isolation, and opportunities for return visits to the country of origin. As heritage languages are not consistently passed on in all migrant families, our aim was to explore the parental perceptions and practices associated with better language outcomes in children.

To clarify the rationale behind this research, we have added the following text at the start of the introduction:

Migration flows cause increasing numbers of children to grow up exposed to two languages, their family’s heritage language (HL) and their host country's majority language (ML). Bilingual children in immigrant families are exposed to diverse language-learning environments and develop widely varying levels of language skills, often with uneven proficiency between their languages [1],[2]. However, acquiring both the heritage and the majority language (i.e., becoming bilingual) is a core component of immigrant children’s adaptive development with many ensuing benefits. HL proficiency has been associated with harmonious family relationships, long-term emotional well-being, stronger identity construction, and psychological adjustment [3–6]. Moreover, combined proficiency in both languages has been linked to abilities that foster academic achievement and support social navigation of youth’s interpersonal worlds [7].

Navigating multiple languages may be demanding. When bilingual developmental difficulties arise, they pose complex challenges for health professionals, educators, and families alike. While numerous standardized tools are available to assess impairment and guide interventions in monolingual contexts, the situation is more complex in cross-cultural and bilingual contexts. The wide variability of language acquisition makes it difficult to distinguish language disorders from language differences, and additional migration-related risks such as discrimination and acculturative stress may further compound the challenges [8]^,^[9]. As highlighted by Stow and Dodd, equitable clinical services require culturally and linguistically appropriate tools and practices to prevent intervention inequities [10]. Identifying factors that may favor or impede bilingual development is therefore essential to help educators, policymakers and health professionals address the specific needs of migrant bilingual children, and optimize interventions to support their language development [11,12].

**Reinforce the theoretical and conceptual approach of the subject matter.**

**Response**: Thank you for your suggestion. We have added the text below to explain our theoretical and conceptual approach.

Our research is grounded in the transcultural clinical approach, the theoretical and methodological foundations of which were built on Devereux’s complementarist paradigm, integrating complementary frames of reference, historically, anthropology and psychoanalysis [34,35]. By transcultural, we refer to the dynamic process of cultural transformation with migration, which combines the loss of existing cultural elements, the borrowing and reappropriation of external cultural traits, and the creation of new cultural forms emerging from cultural interaction [36]. This framework has been further operationalized in transcultural psychotherapy (a second-line method drawing on systemic and psychoanalytic family therapy, narrative therapy, and cultural mediation) that has been developed for migrant patients when standard care proves insufficient [37]. Building on this tradition, our clinical work on language and learning disorders with second-generation migrant children has extended the complementarist paradigm. This work suggests that bilingual acquisition must be understood ecologically—not only from a cognitive viewpoint but also from traditional psychological approaches (parents’ subjective experiences) and socio-cultural perspectives (anthropology, sociolinguistics, etc.) [38]. This original approach resonates to a certain extent with the theoretical underpinnings of several ecological and integrative models, such as the Integrative Risk and Resilience Model of Adaptation in Immigrant-origin Children [39,40] or the concept of familism [30].

**Explain in depth the reason for using mixed methods and their advantages to approach the population of interest.**

**Response**: Thank you for your question.

We wish to clarify that this study does not employ a mixed-methods approach in the strict sense, as it does not include a qualitative analysis of the corpus. Nevertheless, the study design was inspired by the mixed-methods framework. We chose this approach as the most appropriate for capturing subjective phenomena, particularly parental representations of family transmission. This approach allowed parents to voice their feelings and opinions—to explore what they think they do and how they think about their roles in cultural and linguistic transmission. Qualitative data enabled access to these representations in a more nuanced and open-ended way than standardized questionnaires, which, to the best of our knowledge, did not exist for this purpose when we planned this study.

The second phase involved quantifying qualitative data, allowing us to test correlation hypotheses based on a sufficiently large sample size. This analytic strategy aligned with our theoretical framework, which considers migration as a transformative experience that shapes family relationships—both locally and transnationally—and involves a process of *transculturation*, through which parents integrate elements of the host culture while maintaining or modifying aspects of their heritage.

We constructed the questionnaire in accordance with this paradigm. Explanatory variables were defined through thematically grouped questions, each designed to explore a specific aspect of parental perception. A scoring system was developed to assess the intensity of investment in familial relationships, cultural affiliations, and perceived life changes since migration.

We have added the following paragraph to the manuscript to explain the reason for using a method inspired by mixed methods:

This study used a cross-sectional design combining standardized language assessments with an innovative approach to capture parental transcultural perceptions. We drew on procedures inspired by mixed-methods designs. This two-step process involved conducting semi-structured interviews with parents and then quantitizing their narrative responses (i.e., transforming them into numerical scores [44]), thereby generating reproducible variables for statistical analysis. The similar strategies applied in health and social sciences to quantify familial relationships [45] inspired the approach used here. The rationale for this approach was twofold. First, interviews allow parents to voice their feelings and opinions, providing access to their subjective experiences of complex transcultural domains in a more open-ended manner than would be possible with a standardized questionnaire; moreover, when we began this study, no standardized questionnaire existed on this subject, nor has any been published since. Second, a quantitative step is required to test associations between transcultural parental factors and children’s receptive and expressive skills in HL and ML. By grounding the construction of quantitative variables in qualitative parental accounts, our design aimed to operationalize complex transcultural constructs and to explore their relationships with children’s bilingual development in ways that reflect the complexity of their language pathways in migration. This strategy aligned with our theoretical framework of complementary perspectives and allowed us to capitalize on the strengths of each tradition—qualitative data offering depth and contextual richness, and quantitative modeling providing statistical robustness and generalizable estimates—to enhance the interpretability of our findings.

**Expand on the profile and experience of those who applied the measurement instruments.**

**Response:**

We thank you for your question. The following text clarifies who administered the measurement tools used in the study:

These tools were administered by 7 professionals: 5 clinical psychologists with 10 years of specialized experience in individual, group, and transcultural therapy; a psycholinguist specialized in bilingualism and language assessment with 20 years of experience; and a child/adolescent psychiatrist with 15 years of specialization in language assessment in bilingual contexts and in transcultural work with migrant children and their families.

This group is part of a transcultural research team that has been working in the Paris region for over thirty years, developing research on language and mental health among migrant children to better understand their specific vulnerabilities and how to provide them with effective care.

**The problem addressed by the study is relevant to improving the functioning of a multicultural society. It would be desirable to delve into children’s health education and literacy as part of their socialization process.**

**Response:** Thank you for your question. We have added a paragraph at the end of the discussion addressing the implications of our research.

Practical implications : Our results point to several practical implications. First, we suggest that parental transcultural perceptions—how parents value migration-related changes, extended family relationships, and intergenerational transmission—play a role in children’s HL acquisition. Extrapolating these results, we propose that in clinical and educational settings, questions about children’s language use should not be limited to assessing developmental milestones, but could also serve as entry points into understanding identity formation and family relationships. Systematically documenting such perceptions could help identify children at risk of HL erosion and guide more tailored support. Acknowledging transcultural factors that support language development may help families and educators sustain and validate home-based practices as well as contribute to reducing the stigma often attached to minority languages.

Our findings also have implications for children’s health education: supporting HL alongside ML may strengthen health literacy by enabling children to navigate multiple cultural contexts, communicate effectively with both family and healthcare providers, and integrate health knowledge into daily life. Ultimately, rather than viewing multilingualism as a barrier, this approach reframes it as a resource to be nurtured—for the benefit of children, families, and society.

**It is a good study, and I suggest improving the manuscript before sending it to other reviewers. 1) to delve into the theories behind the concepts used. Concepts such as inherited language, extended family relationships and transculturality**.

**Response:** Thank you very much for your interest and your encouraging review. We appreciate your suggestion and have now added details on inherited language, extended family relationships, and transculturality in the introduction.

Within this framework, the term heritage language (HL) is today conceptualized as a nondominant language in a host society that holds personal or cultural significance for the individual (e.g., through family or historical ties), regardless of actual proficiency [41]. Unlike alternative labels such as *L1* or *mother tongue*, the concept of HL shifts the focus from linguistic dimension to a symbolic one; HLs are conceptualized as embodiments of community traditions and identities, with each HL a critical medium for transmitting collective memories, values, and cultural practices [42]. The concept of heritage explicitly embeds an intergenerational perspective, linking language to family transmission across time. Pragmatically, HL proficiency may be necessary to sustain communication with relatives, particularly those grandparents who do not master their grandchildren’s ML. Relationships with extended family members also broaden the contexts in which the HL is used, enriching children’s HL environment both quantitatively and qualitatively. Beyond these practical reasons, qualitative studies have also shed light on the HL’s emotional aspects. For instance, some authors, finding that families express a strong emotional connection to the HL, have proposed that HL is valued for its symbolic maintenance of family bonds and cultural identity [43]. Affective ties with extended kin [30] may lead migrants to make a significant effort to provide crucial support for HL use and for its transmission to their children.

By transcultural, we refer to the dynamic process of cultural transformation with migration, which combines the loss of existing cultural elements, the borrowing and reappropriation of external cultural traits, and the creation of new cultural forms emerging from cultural interaction [36].

**I suggest to the authors that in their methods they should make explicit the justification of: why is it sufficient to study population with three languages -Arabic, Tamil and Soninke-, is it sufficient to consider them representative of transculturality?**

**Response:** Thank you for your question. We have added a paragraph clarifying how the three contexts provide a rationale for adopting a transcultural approach.

The rationale for the selection of this population was to maximize cultural diversity through a strategic yet pragmatic selection of three contexts, focusing on prevalent migrant groups in the region spanning three distinct linguistic families and migration histories. These groups reflect major migration streams to France—North Africa and the Middle East, South Asia, and Sub-Saharan West Africa—whose languages belong to three genealogically unrelated families: Semitic (Maghrebi Arabic), Dravidian (Tamil), and Niger-Congo (Soninke). Maghrebi Arabic families are embedded in a post-colonial labor migration context, with parents typically bilingual but facing stigmatization. Tamil families represent a recent migration linked to the Sri Lankan civil conflict, sustained by strong community practices of language and cultural transmission. Soninke families, largely from West Africa, migrated for economic reasons; Soninke is primarily oral and less institutionalized in written form: it is maintained in France through solidarity networks and ritual events. While neither an exhaustive nor a representative sample of all migrant populations, these groups together constitute a clinically relevant sample whose heterogeneity strengthens the ecological and analytical validity of the study.

**Authors are required to justify the use of measurement instruments and how they were planned to be used with the information collected from the interviews; as well as extending the explanation of the procedure for interpreting numerical data in the light of qualitative data obtained, and vice versa.**

**Response**: Thank you for your question. I hope this response will clarify and adequately address your concerns.

We deemed it necessary to draw on parents’ narratives to better capture their perceptions regarding intergenerational transmission, experiences of change through migration, and extended-family relationships. These dimensions, which stem directly from our theoretical framework, required a more open-ended exploration than any currently existing standardized questionnaire would have allowed.

From the outset, the semi-structured interview guide and the rating grids were conceived to be used together: the guide was designed to elicit parental narratives, which in turn we planned to quantify by using Likert-type rating grids that would transform parents’ perceptions into quantitative variables suitable for the statistical phase.

However, our design does not constitute a mixed-methods approach in the strict sense, since we did not conduct a separate, inductive qualitative analysis of the corpus before the quantitative phase. That is why we wrote that we “drew on procedures inspired by mixed-methods designs.” After the quantitative phase, we did marginally revisit the corpus from the interviews to contextualize the statistical results (for example, the negative link between extended-family perceptions and HL skills), but these observations do not constitute a genuine qualitative analysis. We plan to conduct a full qualitative component in future research, for example through in-depth interviews with families reporting very positive perceptions of extended-family relationships, to shed further light on such associations.

To clarify these points, the Methods section of the revised manuscript now contains an expanded description of the design process. We have also added the following statement to the Discussion (lines 498-500, p. 22): “Future studies should refine these measures and combine them with in-depth qualitative analyses among families with highly positive perceptions to better understand this counterintuitive result.”

**Authors are required to clarify statements such as: 374-376: “Several hypotheses may shed light on the negative relationship between perceptions of extended-family relationships and HL skills. We cannot exclude possible flaws in measuring perceptions of extended-family relationships or failure to adjust for confounding variables”. The above, how it affects the achievement of the study objective reported by the authors.**

**Response:**

We did not express ourselves well in these lines, where we meant to say we could not rule out either possible gaps in our assessment of these perception or a failure to adjust for confounding variables. We therefore deleted this sentence. Specifically we meant that the measure did not collect information about the language used in and the frequency of these conversations with the children. Furthermore your comment prompted us to describe these hypotheses in more detail. We have now used these hypotheses to enrich the interpretation of this counterintuitive result. All of these points are all covered in the text below, which has been added to the text. Thank you for this question.

Several hypotheses may shed light on the negative association between positive perceptions of extended-family relationships and children’s HL skills. When parents receive strong affective support from their extended family, the symbolic function of HL as the primary link to cultural identity may become less central within the nuclear family, diverting parental investment away from HL and reducing pressure on the child to maintain it. Another related explanation is that extended-family members living in France may interact mainly in French, so that supportive ties increase children’s ML rather than HL exposure. A further possibility is that very positive perceptions of extended-family relationships concern geographically distant relatives—a frequent situation in migration contexts—where distance fosters idealization without providing effective HL interactions. The latter two interpretations point to gaps in our instrument, which captures the valence of family ties but neither the frequency nor the language used in these conversations. Future studies should refine these measures and combine them with in-depth qualitative analyses among families with highly positive perceptions to better understand this counterintuitive result.

**Responses to reviewer 1**

**Reviewer #1**: **Reviewer Comments**

**Though timely and relevant, the manuscript suffers from major conceptual and methodological problems that severely restrict its contribution. That is, this study lacks a strong theoretical basis.**

**The key constructs--migration-related changes, extended-family relationships, and transgenerational transmissions--are not defined or theoretically grounded in existing transcultural or socio-linguistic literature. Thus, the framing feels vague and inconsistent throughout**.

**Response:** We thank you for your comment. We have added text to ground theoretically our research in the introduction.

Our research is grounded in the transcultural clinical approach of Georges Devereux ~~(1978),~~ who emphasized the universality of psychic processes as uniquely expressed through culture and language. This dual perspective is essential to understanding the migrant experience, which involves complex psychic work, identity reconfiguration, and evolving cultural affiliations—both retained from the country of origin and adopted in the host country. Parenthood in migration entails navigating these transcultural encounters, balancing losses—such as close family ties and heritage language use—with gains from the host country, including new ideas, experiences, and values. Migrant parents operate in multilingual environments, striving to maintain inherited languages while acquiring the host country’s dominant language. Growing up away from extended family adds challenges, as these relationships provide emotional, educational, and cultural support. Parents must forge new ties across distance, and even when reunited, new forms of family life emerge from the transcultural experience.

We have accordingly included the text below to the introduction:

Our research is grounded in the transcultural clinical approach, the theoretical and methodological foundations of which were built on Devereux’s complementarist paradigm, integrating complementary frames of reference, historically, anthropology and psychoanalysis [34,35]. By transcultural, we refer to the dynamic process of cultural transformation with migration, which combines the loss of existing cultural elements, the borrowing and reappropriation of external cultural traits, and the creation of new cultural forms emerging from cultural interaction [36]. This framework has been further operationalized in transcultural psychotherapy (a second-line method drawing on systemic and psychoanalytic family therapy, narrative therapy, and cultural mediation) that has been developed for migrant patients when standard care proves insufficient [37]. Building on this tradition, our clinical work on language and learning disorders with second-generation migrant children has extended the complementarist paradigm. This work suggests that bilingual acquisition must be understood ecologically—not only from a cognitive viewpoint but also from traditional psychological approaches (parents’ subjective experiences) and socio-cultural perspectives (anthropology, sociolinguistics, etc.) [38]. This original approach resonates to a certain extent with the theoretical underpinnings of several ecological and integrative models, such as the Integrative Risk and Resilience Model of Adaptation in Immigrant-origin Children [39,40] or the concept of familism [30].

We have also modified the description of the study design in the Materials and Methods section:

This study used a cross-sectional design combining standardized language assessments with an innovative approach to capture parental transcultural perceptions. We drew on procedures inspired by mixed-methods designs. This two-step process involved conducting semi-structured interviews with parents and then quantitizing their narrative responses (i.e., transforming them into numerical scores [44]), thereby generating reproducible variables for statistical analysis. The similar strategies applied in health and social sciences to quantify familial relationships [45] inspired the approach used here. The rationale for this approach was twofold. First, interviews allow parents to voice their feelings and opinions, providing access to their subjective experiences of complex transcultural domains in a more open-ended manner than would be possible with a standardized questionnaire; moreover, when we began this study, no standardized questionnaire existed on this subject, nor has any been published since. Second, a quantitative step is required to test associations between transcultural parental factors and children’s receptive and expressive skills in HL and ML. By grounding the construction of quantitative variables in qualitative parental accounts, our design aimed to operationalize complex transcultural constructs and to explore their relationships with children’s bilingual development in ways that reflect the complexity of their language pathways in migration. This strategy aligned with our theoretical framework of complementary perspectives and allowed us to capitalize on the strengths of each tradition—qualitative data offering depth and contextual richness, and quantitative modeling providing statistical robustness and generalizable estimates—to enhance the interpretability of our findings.

We list below the questions used to define each variable:

Perception of migration-related change (MRC)

What do you think of the changes in your life since you arrived in France?

Let the parent talk and then have him/her specify the items considered important

You said: " _ _ _ _ _ ." Can you be more specific? Can you give me an example that illustrates what you just said?

Perception of extended-family relationships (EFR)

*What members of your family do you see? Parents, grandparents, brothers and sisters, aunts and uncles, cousins etc.)? You may take your time to answer to answer.*

Reminders:

*• Do you see your family here?*

*• Do you see your family in your native country?*

*•* If the person answered that she sees no one: *Do you get any news from or about your family?*

*How are things with your family: parents, grandparents, brothers and sisters, aunts and uncles, cousins, etc.)? Can you tell me about your relationships with your family?*

Let them talk and then:

*Are things as you would like them to be?*

Reminder: *You said: " _ _ _ _ _ ." Can you be more specific? Can you give me an example that illustrates what you just said?*

Perception of transgenerational transmission: insertion in the family line (PTT)

*How is your relationship with your child? You can take your time to answer*

Reminder: *You said: " _ _ _ _ _ ." Can you tell us about this relationship? Can you give me an example that illustrates what you just said?*

*What has your child taken from his maternal or paternal grandparents? (Physical resemblance, or similar character or values…)*

*What have you taken from your parents that you have taught your child?*

*How did you choose his or her first name?*

*Does the relationship you have with your child resemble the one you had with your parents?*

**A quantitative analysis of open-ended interview data raises serious concerns about its validity and reliability. Evidence of psychometric testing or inter-rater agreement is not available. Inter-rater reliability should be reported.**

**Moreover, the reduction of moods and impressions to binary categories distorts complex cultural experiences.**

**Response:** We thank you for your question and we have added the following text to give more details about the inter-rater agreement.

The instrument we developed has two components: the first a semistructured interview guide for the investigators who interviewed parents about transcultural/familial factors; and the second a Likert-type numerical value scoring grid for each factor, to be used by the raters to quantitize the qualitative corpus made up of parents’ narrative answers. Supplementary File S1 presents the tool development process and testing phases, while File S2 provides excerpts (translated into English) from the semi-structured interview guide used by the investigators to interview parents about transcultural factors, as well the Likert-type numerical scoring grids used by raters to quantitize the qualitative corpus for each factor. In practice, semistructured interviews were conducted in the language chosen by the parents, audio-recorded, translated (when necessary), and then fully transcribed. Next two researchers (not the same pair of raters for all subjects) independently used the corresponding scoring grids to rate the intensity of each factor for each subject. Value range scores obtained by quantization had direction and magnitude representing the intensity of the factor (ranging from -5 to 5): the more positively the parent perceived a factor, the higher the score. For each variable, anchor points were operationalized by a specific statement that best exemplified that specific level of intensity. For example, concerning the factor of transgenerational transmission, the statement descriptive of a rating of -5 is: “*the subject is at odds with transmission, which may be rejected or represent an obstacle for the subject. The question of transmission can be associated with insecurity and abandonment. Transgenerational transmission is not encouraged and is blocked*.” The statement descriptive of a rating of 5 (positive) was “*the participant values this transmission, writing him- or herself and the child in a filiation; cultural transmission appears direct and intense, with strong intention”.* Supplementary File S3 provides excerpts of the semi-structured interview guide that the investigators used to interview parents about transcultural factors, as well as the Likert-type numerical scoring grids used by raters to quantitize the qualitative corpus for each factor.

These tools were administered by 7 professionals: 5 clinical psychologists with 10 years of specialized experience in individual, group, and transcultural therapy; a psycholinguist specialized in bilingualism and language assessment with 20 years of experience; and a child/adolescent psychiatrist with 15 years of specialization in language assessment in bilingual contexts and in transcultural work with migrant children and their families. Two raters independently applied the scoring for each factor, initially blinded to the other rater's score. The intraclass correlation (ICC), that is, the interrater agreement measured with random effects model), was 0.65 (95%CI 0.53-0.75) for transgenerational transmission, 0.77 (95%CI, 0.69-0.83) for extended-family relationships, and 0.84 (95%CI 0.77-0.89) for migration-related change. Then the two discussed and recalibrated each score in case of divergence. When discrepancies could not be resolved, the data were reanalyzed by the entire expert group. The final score was the consensus obtained.

Importantly, our aim was to quantify the perceived intensity and valence (positive or negative) of investment in transgenerational transmission, family relationships, and change. This approach focuses on the subjective intensity rather than the complexity or richness of the narratives. To minimize measurement bias, we implemented independent double ratings, complemented by consensus discussions within a dedicated working group.

**The authors draw strong conclusions from weak associations. Some of the findings are marginally significant or inconsistent, yet interpretable. For instance, the unexpected negative link between positive extended-family perceptions and heritage language ability is scantily explained, appearing rather speculative.**

**Response:**

Thank you for your comment. To provide a more accurate interpretation of the negative association observed between the perception of extended family relationships and heritage language skills, we have added the following explanation to the manuscript.

Several hypotheses may shed light on the negative association between positive perceptions of extended-family relationships and children’s HL skills. When parents receive strong affective support from their extended family, the symbolic function of HL as the primary link to cultural identity may become less central within the nuclear family, diverting parental investment away from HL and reducing pressure on the child to maintain it. Another related explanation is that extended-family members living in France may interact mainly in French, so that supportive ties increase children’s ML rather than HL exposure. A further possibility is that very positive perceptions of extended-family relationships concern geographically distant relatives—a frequent situation in migration contexts—where distance fosters idealization without providing effective HL interactions. The latter two interpretations point to gaps in our instrument, which captures the valence of family ties but neither the frequency nor the language used in these conversations. Future studies should refine these measures and combine them with in-depth qualitative analyses among families with highly positive perceptions to better understand this counterintuitive result.

**The study used a small, non-random sample from one urban area in France, of which only participants from three language groups could be counted against the accolade for being "multicultural**".

Thank you for your question. While our study involved a relatively small, non-random sample drawn from a single urban area in France we deliberately included participants from three distinct linguistic and cultural groups—Tamil, Maghrebi Arabic, and Soninke—each representing different migration histories and sociocultural backgrounds. This diversity allows us to explore migration and transmission processes across multiple cultural frameworks, enriching the analysis despite the sample size and geographic limitation.

We have added this text in the manuscript:

The rationale for the selection of this population was to maximize cultural diversity through a strategic yet pragmatic selection of three contexts, focusing on prevalent migrant groups in the region spanning three distinct linguistic families and migration histories. These groups reflect major migration streams to France—North Africa and the Middle East, South Asia, and Sub-Saharan West Africa—whose languages belong to three genealogically unrelated families: Semitic (Maghrebi Arabic), Dravidian (Tamil), and Niger-Congo (Soninke). Maghrebi Arabic families are embedded in a post-colonial labor migration context, with parents typically bilingual but facing stigmatization. Tamil families represent a recent migration linked to the Sri Lankan civil conflict, sustained by strong community practices of language and cultural transmission. Soninke families, largely from West Africa, migrated for economic reasons; Soninke is primarily oral and less institutionalized in written form: it is maintained in France through solidarity networks and ritual events. While neither an exhaustive nor a representative sample of all migrant populations, these groups together constitute a clinically relevant sample whose heterogeneity strengthens the ecological and analytical validity of the study.

**Cross-sectional design does not allow for any sort of causal inference, even if the authors' wording hints at that**.

**Response:** Thank you for your comment. We agree that a cross-sectional design does not permit causal inference. However, we did observe an association, which we believe should be explored further in a longitudinal study.

**Thus, the authors need to clarify how language proficiency was assessed and ensure that a balance is made in the heritage/majority language mix**. **The authors should also clarify how language proficiency was assessed and ensure the balance between heritage and majority languages.**

**Response:** Thank you for your question~~.~~

For heritage language proficiency, we employed the Avicenne ELAL, a translinguistic and transcultural tool specifically designed to evaluate basic language knowledge transmitted within the family environment (Rezzoug et al., 2018). The test comprises three subscales: comprehension skills, production skills, and narrative skills. Each subtest targets translinguistic concepts considered language universals, that is, features present across a wide range of languages. Given that the instrument was originally developed for young children, it focuses on notions typically acquired early through family interactions.

Children’s performance is summarized by a competency score on each subscale.

To assess proficiency in French, we used the NEEL (Chevrie-Muller & Plaza, 2002), a standardized tool widely utilized by speech therapists in France for evaluating oral language skills.

To ensure comparability, we selected NEEL subscales aligned with those assessed in the Avicenne ELAL.

We chose to examine the two languages separately; however, in the models, we systematically controlled for proficiency in the other language to account for language balance

**The statistical approach would need to be revised, probably better through latent constructs or stronger methodologies overall. The statistical approach should be reconsidered, possibly using latent constructs or more robust techniques. Inter-rater reliability should be reported.**

**Response:**

We appreciate the recommendation regarding the statistical methodology. Future analyses could benefit from employing latent construct modeling or more robust statistical techniques to better capture the complexity of transcultural factors and bilingual proficiency.

Regarding interrater reliability, as detailed elsewhere in the manuscript, intraclass correlation coefficients indicated moderate to good agreement across transcultural perception ratings (ICC ranging from 0.65 to 0.84), thus supporting the reliability of our qualitative data quantification.

**Finally, practical implications for clinicians and educators should be further spelled out.**
**Finally, practical implications for clinicians and educators should be made more concrete.**

**Response:** We thank you for this valuable suggestion. In response, we have added a section at the end of the discussion addressing the practical implications of our findings.

Practical implications : Our results point to several practical implications. First, we suggest that parental transcultural perceptions—how parents value migration-related changes, extended family relationships, and intergenerational transmission—play a role in children’s HL acquisition. Extrapolating these results, we propose that in clinical and educational settings, questions about children’s language use should not be limited to assessing developmental milestones, but could also serve as entry points into understanding identity formation and family relationships. Systematically documenting such perceptions could help identify children at risk of HL erosion and guide more tailored support. Acknowledging transcultural factors that support language development may help families and educators sustain and validate home-based practices as well as contribute to reducing the stigma often attached to minority languages. Our findings also have implications for children’s health education: supporting HL alongside ML may strengthen health literacy by enabling children to navigate multiple cultural contexts, communicate effectively with both family and healthcare providers, and integrate health knowledge into daily life. Ultimately, rather than viewing multilingualism as a barrier, this approach reframes it as a resource to be nurtured—for the benefit of children, families, and society.

**RESPONSES TO REVIEWER 2**

**Reviewer #2: Reviewer Comments**
Overall Evaluation:
This manuscript presents original research exploring the relationship between parental transcultural perceptions and bilingual language development among children of migrant families in France. The topic is timely, relevant, and grounded in a strong conceptual and methodological framework. The study is well-designed, the analyses are technically sound, and the conclusions are data-driven. However, some areas would benefit from minor clarification and improvement, especially in reporting and framing.

Evaluation by Criteria
1. Originality and Novelty -Meets standard.
The study addresses a novel question by linking transcultural parental perceptions with heritage and majority language development. Its innovative and well-executed approach to " quantifying " qualitative data for regression analysis is also noteworthy.

2. Prior Publication- Meets standard.
The results have not been published elsewhere, as declared in the manuscript.

3.Technical Rigor and Detail- Meets standard.
The methodology—including sample selection, instruments (ELAL and N-EEL), and statistical modeling—is clearly described and appropriate. The use of multiple adjusted regression models strengthens the findings.

**Recommendation:** **Consider elaborating on the construct validity and inter-rater reliability of the scoring system used to "quantitize" parental perceptions, as this is critical to the integrity of the analysis.**

**Response:** We are grateful for your thoughtful comments and the encouragement they provide. We have revised the methodology section to clarify the requested aspects.

We developed a transcultural perception measurement tool in collaboration with experts from transcultural clinics, each with more than a decade of experience working with first- and second-generation migrants and familiarity with the parents’ regions of origin. The tool comprises: (i) a semistructured interview guide for investigators to explore transcultural and familial factors with parents; and (ii) a Likert-type scoring grid enabling raters to quantitatively assess the qualitative narrative data.

Scores ranged from -5 to 5, reflecting the direction and intensity of each factor, with higher scores indicating a more positive parental perception. To ensure high inter-rater reliability, anchor points were defined by specific statements exemplifying each intensity level. For instance, for the transgenerational transmission factor (TGT), a rating of -5 corresponds to: “The subject is at odds with transmission, which may be rejected or viewed as an obstacle, associated with insecurity and abandonment; transgenerational transmission is discouraged or blocked.” Conversely, a rating of 5 indicates: “The participant highly values transmission, situating themselves and their child within a filiation; cultural transmission is direct, intense, and intentional.”Transcultural qualitative data were collected through semistructured interviews with participating parents, conducted in the language of their choice. Interviews were audio-recorded, translated when necessary, and fully transcribed. Subsequently, two independent researchers rated the intensity of each transcultural factor per participant using predefined scoring grids; pairs of raters varied across subjects. Interrater reliability, assessed via intraclass correlation coefficients (ICC) using a random effects model, indicated moderate to good agreement: the ICC for transgenerational transmission (TGT) was 0.65 (95% CI 0.53–0.75), for extended-family relationships (EFR) 0.77 (95% CI 0.69–0.83), and for migration-related change (MRC) 0.84 (95% CI 0.77–0.89). Any discrepancies between raters were resolved through discussion within the expert group (see Appendix 1), and consensus ratings were assigned.

Score distributions reflected predominantly positive parental perceptions across all transcultural factors: MRC (median = 3.0, range -4 to 5), EFR (median = 4.2, range -3 to 5), and TGT (median = 3.8, range -1 to 5).

It is important to note that the aim was to quantify the *perception of investment* in transgenerational transmission, family relationships, and migration-related change. Therefore, even small changes could be perceived with strong positive or negative intensity. Our rating approach focused on the valence and intensity rather than the richness or complexity of narratives. To mitigate measurement bias, we implemented a consensus-based expert working group and performed independent double ratings.

We have added the following text in the Methods section to clarify our conceptual framework:

Study design

This study used a cross-sectional design combining standardized language assessments with an innovative approach to capture parental transcultural perceptions. We drew on procedures inspired by mixed-methods designs. This two-step process involved conducting semi-structured interviews with parents and then quantitizing their narrative responses (i.e., transforming them into numerical scores [44]), thereby generating reproducible variables for statistical analysis. The similar strategies applied in health and social sciences to quantify familial relationships [45] inspired the approach used here. The rationale for this approach was twofold. First, interviews allow parents to voice their feelings and opinions, providing access to their subjective experiences of complex transcultural domains in a more open-ended manner than would be possible with a standardized questionnaire; moreover, when we began this study, no standardized questionnaire existed on this subject, nor has any been published since. Second, a quantitative step is required to test associations between transcultural parental factors and children’s receptive and expressive skills in HL and ML. By grounding the construction of quantitative variables in qualitative parental accounts, our design aimed to operationalize complex transcultural constructs and to explore their relationships with children’s bilingual development in ways that reflect the complexity of their language pathways in migration. This strategy aligned with our theoretical framework of complementary perspectives and allowed us to capitalize on the strengths of each tradition—qualitative data offering depth and contextual richness, and quantitative modeling providing statistical robustness and generalizable estimates—to enhance the interpretability of our findings.

Factors related to transcultural perception:

The instrument we developed has two components: the first a semistructured interview guide for the investigators who interviewed parents about transcultural/familial factors; and the second a Likert-type numerical value scoring grid for each factor, to be used by the raters to quantitize the qualitative corpus made up of parents’ narrative answers. Supplementary File S1 presents the tool development process and testing phases, while File S2 provides excerpts (translated into English) from the semi-structured interview guide used by the investigators to interview parents about transcultural factors, as well the Likert-type numerical scoring grids used by raters to quantitize the qualitative corpus for each factor. In practice, semistructured interviews were conducted in the language chosen by the parents, audio-recorded, translated (when necessary), and then fully transcribed. Next two researchers (not the same pair of raters for all subjects) independently used the corresponding scoring grids to rate the intensity of each factor for each subject. Value range scores obtained by quantization had direction and magnitude representing the intensity of the factor (ranging from -5 to 5): the more positively the parent perceived a factor, the higher the score. For each variable, anchor points were operationalized by a specific statement that best exemplified that specific level of intensity. For example, concerning the factor of transgenerational transmission, the statement descriptive of a rating of -5 is: “*the subject is at odds with transmission, which may be rejected or represent an obstacle for the subject. The question of transmission can be associated with insecurity and abandonment. Transgenerational transmission is not encouraged and is blocked*.” The statement descriptive of a rating of 5 (positive) was “*the participant values this transmission, writing him- or herself and the child in a filiation; cultural transmission appears direct and intense, with strong intention”.* Supplementary File S3 provides excerpts of the semi-structured interview guide that the investigators used to interview parents about transcultural factors, as well as the Likert-type numerical scoring grids used by raters to quantitize the qualitative corpus for each factor.

These tools were administered by 7 professionals: 5 clinical psychologists with 10 years of specialized experience in individual, group, and transcultural therapy; a psycholinguist specialized in bilingualism and language assessment with 20 years of experience; and a child/adolescent psychiatrist with 15 years of specialization in language assessment in bilingual contexts and in transcultural work with migrant children and their families. Two raters independently applied the scoring for each factor, initially blinded to the other rater's score. The intraclass correlation (ICC), that is, the interrater agreement measured with random effects model), was 0.65 (95%CI 0.53-0.75) for transgenerational transmission, 0.77 (95%CI, 0.69-0.83) for extended-family relationships, and 0.84 (95%CI 0.77-0.89) for migration-related change. Then the two discussed and recalibrated each score in case of divergence. When discrepancies could not be resolved, the data were reanalyzed by the entire expert group. The final score was the consensus obtained.

4. Conclusions and Data Support- Meets standard.
Conclusions are well-grounded in the data, clearly acknowledging limitations such as the cross-sectional design and lack of causal inference.

5.Clarity and Use of English- Meets standard.
The article is written in clear and intelligible English. Minor copyediting could further improve flow, but no major issues are present.

6.Ethical Standards- Meets standard.
Ethics approval was obtained from an appropriate IRB (North University Hospitals of Paris). Informed consent procedures are properly described.

7.Research Integrity- Meets standard.
The study adheres to good research practices, and there are no signs of data manipulation or undue bias.

8.Reporting Guidelines and Data Availability- Partially meets standard.
The authors note that data cannot be shared due to French privacy laws. While this may be valid, PLOS ONE requires full transparency.
Recommendation: The authors should clarify what aspects of the data are restricted and whether anonymized or partial datasets (e.g., coded scores) could be made available under controlled access or through a trusted repository.

**Final Recommendation:
Minor Revision
The manuscript strongly contributes to the literature on bilingualism and migration studies. I recommend acceptance pending minor revision, specifically**

**To clarify the data availability constraints**

The dataset underlying this article contains sensitive health and migration-related information, which is considered highly protected under French regulations. For this reason, the data cannot be made publicly available.

We have now arranged with the competent authorities that access to de-identified individual-level numerical data can be granted upon request, subject to approval by the relevant ethics and scientific committees and after the signature of a data transfer agreement. Requests should be addressed to Dalila Rezzoug, mail: dalila.rezzoug@aphp.fr.

**Add more detail regarding the quantification of transcultural factors.**

**Response:** Thank you for your request for clarification. We have supplemented the information on the quantification of transcultural data, emphasizing the value of our methodology in constructing transcultural explanatory variables.

We chose an approach inspired by mixed-methods studies as most appropriate for capturing subjective phenomena, particularly parental representations of family transmission. This approach allowed us to give voice to parents—to explore what they say they do and how they think about their roles in cultural and linguistic transmission. Qualitative data enabled access to these representations in a more nuanced and open-ended way than standardized questionnaires, which, to our knowledge, do not yet exist for this purpose.

The second phase involved quantifying qualitative data, allowing us to test correlation hypotheses based on a sufficiently large sample size. This analytical strategy aligned with our theoretical framework, which considers migration as a transformative experience that shapes family relationships—both locally and transnationally—and involves a process of *transculturation*, through which parents integrate elements of the host culture while maintaining or modifying aspects of their heritage.

We constructed the questionnaire in accordance with this paradigm. Explanatory variables were defined through thematically grouped questions, each designed to explore a specific aspect of parental perception. A scoring system was developed to assess the intensity of investment in familial relationships, cultural affiliations, and perceived life changes since migration.
